# Male Hypogonadism and Osteoporosis: The Effects, Clinical Consequences, and Treatment of Testosterone Deficiency in Bone Health

**DOI:** 10.1155/2017/4602129

**Published:** 2017-03-16

**Authors:** Gary Golds, Devon Houdek, Terra Arnason

**Affiliations:** ^1^Department of Medicine, University of Saskatchewan, Saskatoon, SK, Canada S7N 0W8; ^2^Division of Endocrinology and Metabolism, Department of Medicine, University of Saskatchewan, Saskatoon, SK, Canada S7N 0W8

## Abstract

It is well recognized that bone loss accelerates in hypogonadal states, with female menopause being the classic example of sex hormones affecting the regulation of bone metabolism. Underrepresented is our knowledge of the clinical and metabolic consequences of overt male hypogonadism, as well as the more subtle age-related decline in testosterone on bone quality. While menopause and estrogen deficiency are well-known risk factors for osteoporosis in women, the effects of age-related testosterone decline in men on bone health are less well known. Much of our knowledge comes from observational studies and retrospective analysis on small groups of men with variable causes of primary or secondary hypogonadism and mild to overt testosterone deficiencies. This review aims to present the current knowledge of the consequences of adult male hypogonadism on bone metabolism. The direct and indirect effects of testosterone on bone cells will be explored as well as the important differences in male osteoporosis and assessment as compared to that in females. The clinical consequence of both primary and secondary hypogonadism, as well as testosterone decline in older males, on bone density and fracture risk in men will be summarized. Finally, the therapeutic options and their efficacy in male osteoporosis and hypogonadism will be discussed.

## 1. Introduction

Hypogonadism in adults is a well-acknowledged cause of overall bone loss and a contributor to the development of secondary osteoporosis. In female osteoporosis, bone loss has been well correlated to the declines in estrogen, generally following menopause. In normal male aging, a gradual decline in testosterone production can also occur with some older men developing late onset hypogonadism which is marked by significantly low testosterone levels and concurrent symptoms of hypogonadism. It seems reasonable to anticipate that low bioavailable levels of testosterone in aging men, similar to the decreased estrogen levels in menopause, would correlate to a loss in bone mineral density (BMD) and an increase in fracture risk. As expected, male hypogonadism is correlated with losses in bone quality, yet unexpectedly, the connection is not tightly dependent on testosterone levels. In general, it is not common to consider estrogen (E2) levels in men as contributing to bone health in men, yet the relative bioavailable E2 levels have the strongest correlation with maintenance of bone density. Nonetheless, hypogonadal men treated with testosterone do have statically significant gains in bone density over relatively short periods of time. To date, there have been limited studies, either clinical, in vitro, or in vivo, that specifically address the normal physiology of testosterone on the bone and the effect on bone quality in adult males in the presence of hypogonadism. As such, this review will focus on answering key questions regarding male hypogonadism as well as highlighting areas requiring further research and exploration. Specifically, we will address the following: What direct and indirect effects do testosterone, gonadotropins, and related hormones have on bone metabolism; what is the prevalence of male osteoporosis and what proportion arises from hypogonadism; what are the clinical consequences of hypogonadism on bone quality and fracture risk; and how effectively can hormonal and/or other therapies reverse bone loss in men?

## 2. Definition and Epidemiology of Male Osteoporosis

### 2.1. How Do We Diagnose/Define Osteoporosis in Males?

Osteoporosis, as described by the World Health Organization (WHO) since 1994, is a condition characterized by low bone mass and microarchitectural bone deterioration that leads to bone fragility and fracture susceptibility [1]. In 1994, the WHO established diagnostic criteria for osteoporosis in postmenopausal females based on their bone mineral density (BMD) readings. They defined osteoporosis as the category in which individuals have a BMD standard deviation (SD) measurement, referred to as the T-score, of 2.5 or less below the norm established from their young adult female Caucasian reference population. Since then, the diagnostic criterion has been further refined by the WHO Collaborating Centre in 2007 who advocated for the comparative reference standard to be the BMD measured at the femoral neck (FN) using dual-energy X-ray absorptiometry (DXA) and in comparison to Caucasian women aged 20–29 within the NHANES III database [2].

This updated reference standard has been accepted by a number of international organizations and authorities [3–6], yet many recognize that this Caucasian and female-only reference population is less than ideal for different ethnicities and male gender. Regarding male osteoporosis specifically, some organizations, including those in Canada, the USA, and Europe, have adopted the use of gender-specific reference populations [7, 8]. Ideally, in order to capture and acknowledge the differences in the peak BMD reference that occurs between males and females, the male peak BMD reference should be higher than that of females. Therefore, using a female-only reference underestimates the degree of male bone loss and by extension the diagnosis of osteoporosis [9–11].

However, studies have illustrated that despite this underestimation of the degree of deviation males have from a male reference norm, the fracture risk is the same between males and females for a given DXA BMD value and thus distinguishing the SD from a gender-specific reference is perhaps of little clinical significance [12–15]. Furthermore, the importance of BMD measurements has decreased as emphasis has shifted away from BMD measurements for diagnosis, and more towards the clinically significant fracture risk assessment, of which BMD measurements are only one of many factors that are now considered. For simplicity, it has been advocated by the WHO and others to use the traditional standard female-only reference population. Thus, regardless of whether one uses a female or male reference population, the traditional diagnostic category of BMD T-score SD ≤ −2.5 and between ≤1.0 and ≥2.5 is universally used to define osteoporosis and osteopenia in males aged ≥ 50, respectively. For individuals under age 50, the BMD Z-score, which uses an age- and a gender-specific reference, is used.

### 2.2. What Is the Prevalence of Male Osteoporosis and Hypogonadal-Related Osteoporosis?

Although the prevalence of osteoporosis amongst males ≥ 50 is significantly lower than the female population, male osteoporosis and osteopenia and its clinical consequences are significant. Though men tend to sustain osteoporotic fractures up to 10 years later in life than women, the mortality and morbidity associated with male hip fractures are higher than that of women, and men with known fragility fractures are less likely to receive treatment as compared to women [16]. Despite this, osteoporosis research has been highly female dominant, but an increasing awareness, insightful research, and greater appreciation of the importance of male bone quality continues to contribute to our understanding of male hypogonadal osteoporosis. The WHO has compiled data on the prevalence of male and female osteoporosis from different epidemiological studies around the world [1]. Their data is derived from the EVOS (European), CaMos (Canada), Rotterdam (Netherlands), Dubbo (Australia), Rochester (USA), and Hiroshima (Japan) studies. The pooled estimated prevalence of male osteoporosis is very low under age 70, yet rises significantly to an estimated prevalence of 22.6% in the very aged (>90 years old). A similar estimated prevalence of female osteoporosis reveals a significantly greater incidence for any age, including those under 70 years old (Table 1).

Epidemiological information on male osteoporosis arising from secondary causes, and male hypogonadism, specifically, is lacking, and therefore, the prevalence of male osteoporosis attributed to hypogonadism is unclear. The studies that have been done are few, involve a small number of centers, and have low patient numbers. The rate of identified secondary male osteoporosis (all causes) or osteoporosis-related fracture has been relatively consistent amongst multiple studies with ~50% having an attributable secondary cause for their osteoporosis [17–20]. Those few studies that reported the secondary causes for male osteoporosis have found rates ranging from 16 to 30% for hypogonadism as the attributable cause [18–22]. These small studies illustrate the high degree of identifiable causes of secondary male osteoporosis and the significant amount of hypogonadal-related male osteoporosis and osteoporosis-related fractures. These studies are however limited by their small sizes and fraught with all kinds of potential bias. Large studies are needed to better quantify the prevalence of secondary causes of male osteoporosis/osteopenia and osteoporosis-related fractures.

### 2.3. Are There Differences in the Architecture or Structure of Bone between Genders and Does This Impact Fracture Risk?

Given that the peak bone mass in males is higher than females, it is reasonable to expect that for a given BMD, males have a greater risk for fracture, as they have deviated further from their peak density. Yet, as mentioned above, studies have shown that for a given DXA BMD score, the fracture risk between genders is indistinguishable. There is evidence that there are a number of characteristic differences between male and female bone architecture and structure that offer a potential advantage to males that may explain their reduced fracture susceptibility [23, 24]. It is well documented that peak bone mass is greater in males due, in part, to simply having larger bones on average [23, 24]. Other studies have also shown that the cross-sectional diameter of vertebrae and femoral necks are larger in males, and that this is associated with greater bone strength, as compared to that in females [25–30]. Analysis of men discovered to have incidental radiographic fragility fractures has found a convincing link to these individuals having lower bone mass and decreased bone cortical thickness; these correlate with fracture risk independent of BMD [31].

It is also interesting to consider the type of bone loss during normal aging and how it compares between men and women. Healthy men possess a greater maximum degree of periosteal thickening than women that may contribute to the above-mentioned greater cortical thickness and cross-sectional diameter [23, 24, 26, 29, 32]. Therefore, males potentially enter age-related bone loss at an advantage. Interestingly, there are sex-related differences in the rate of age-related adverse effects on trabecular bone, where women lose both periosteal thickening and cortical thickness more rapidly than males [23, 24, 26, 29, 32]. The type of trabecular bone remodeling that occurs with aging also differs between the sexes, as males tend to have trabecular thinning and females tend to lose trabecular connectivity [23]. Therefore, despite the fact that males will have a larger deviation away from their gender-specific reference for any given DXA BMD value, this does not seem to translate into an increased fracture risk, and is perhaps related to the protective benefit of their peak bone characteristics, and different types of bone architectural changes with aging, as compared to females.

## 3. What Is the Normal Physiological Effect of Androgens on Bone Quality in Males?

### 3.1. Physiological Effect of Androgens and Their Receptors on Bone Metabolism

The exact role of testosterone on male bone development and maintenance is still being determined. The androgen receptor has been found to be expressed in osteoblasts (bone deposition), osteoclasts (bone resorption), osteocytes (bone homeostasis), and pluripotent mesenchymal bone marrow stromal cells (progenerators), and it is likely that androgens have a direct role in affecting the function of all of these bone-related cells and overall bone metabolism [33, 34]. Observational studies and case reports in patients with androgen insensitivity syndrome (AIS), where there is a partial or complete lack of androgen receptor signaling [35], have demonstrated reduced BMD in people with AIS, particularly in the lumbar spine and regardless of estrogen replacement [36–39]. This supports a direct role for androgen signaling and testosterone action in bone development and maintenance. Testosterone also contributes to indirect effects on bone through its conversion via aromatase to estrogen [40] as males with aromatase deficiency almost universally present with osteopenia or osteoporosis [41–44], and selective blockade of aromatase activity leads to decreased BMD in men [45, 46]. Taken together, it is likely that testosterone has direct effects on bone quality via the androgen receptor as well as indirect effects via conversion to estrogen by aromatase.

#### 3.1.1. Limitations of Animal Models to Study Androgen Effects on Bone Health

Much of the knowledge available on the molecular and biochemical effects of androgens and estrogens on bone homeostasis comes from mouse models [33, 40]. However, caution must be used when extrapolating these results to humans. First of all, mice and rats lack sex hormone-binding globulin (SHBG) [47], which is the major serum-binding globulin of testosterone and estrogen. SHBG levels regulate the bioavailable levels of these hormones [48] as only estrogen and testosterone that is not bound to SHBG, but rather bound by albumin and other carrier proteins or is circulating freely, are considered available for biological signaling and activity [48]. The importance of SHBG in human bone health has been demonstrated by studies, which show that serum SHBG levels are inversely associated with BMD in both men and women [49, 50]. The lack of SHBG in rodents may also mean that rodents experience greater fluctuations in sex steroid levels, and these fluctuations in themselves may be important in regulating bone metabolism [40]. For example, in humans, parathyroid hormone (PTH) stimulates bone formation when given in a pulsatile fashion, whereas chronic exposure to PTH results in bone resorption. This demonstrates how pulsatile or intermittent surges of hormone levels may have different effects on bone function as compared to a more regulated chronic exposure to these same hormones. The levels of free estrogen in mice are also significantly lower when compared to humans which may suggest that localized metabolism of sex hormones in mice contribute more significantly to bone physiology in murine models as compared to humans. Models of primary hypogonadism utilizing orchiectomy also result in the loss of other gonadal-specific hormones such as inhibin A which may also contribute to bone health [51]. Finally, global knockouts in mice of androgen or estrogen receptors lead to impairment of important negative regulation mechanisms outside of the bone itself which can lead to further derangement of hormone levels [52, 53]. Therefore, to best understand the specific effects of sex steroids on the bone, targeted knockouts in bone-specific cell lines such as osteoblasts, osteoclasts, and osteocytes should be used when trying to determine the role of sex steroids in bone function.

### 3.2. Direct Effects of Testosterone and the Androgen Receptor on Osteoblasts, Osteocytes, and Osteoclasts

Despite their limitations, mouse cell lines have provided insights into the role of testosterone in bone cells by generating cell line-specific knockouts of aromatase and the androgen receptor often by using *Cre-Lox* recombination technology. In osteoblasts, the loss of the androgen receptor resulted in decreases in trabecular bone mass, fewer trabeculae, and an increase in trabecular separation with no effect on cortical bone [54–56]. This suggests that testosterone signaling through the androgen receptor in osteoblasts is important in trabecular but not cortical bone formation. This also suggests an important role of testosterone in contributing to bone strength and fracture risk as trabecular bone is an important determinant to both [57]. The direct role of testosterone in osteoclasts is less well established. We identified a single report using osteoclast-specific androgen receptor knockout mice, which reported no effect on bone mass in either male or female mice; however, full gene knockdown could not be confirmed, leaving these results in question [54]. This result is also somewhat surprising as it has also been found that there are an increased number of osteoclasts in the lumbar spine of these knockout mice [58, 59]. Overall, there needs to be further research done on the exact role of testosterone in osteocyte signaling and function.

Finally, osteocytes also express the androgen receptor and demonstrate increased androgen receptor expression with osteocyte differentiation suggesting a role for testosterone in their function [60]. Murine models with osteocyte-specific androgen receptor knockout have demonstrated decreases in trabecular bone volume and trabeculae number, and these decreases worsened with age [60]. Two additional mouse lines with osteocyte-specific androgen receptor knockout showed the role of the androgen receptor in prevention of age-related trabecular bone loss but not anabolic bone formation [61]. Overall this suggests that the androgen receptor in osteocytes is important for age-related prevention of trabecular bone resorption. In summary, testosterone likely has direct effects via the androgen receptor on osteoblasts by promoting trabecular bone formation and on osteocytes by preventing age-related resorption of trabecular bone. Further studies need to be conducted to fully elucidate any role of the androgen receptor in osteoclast function.

### 3.3. Indirect Effects of Testosterone via Aromatase Conversion to Estrogen

Testosterone can also have indirect effects in the body through its conversion to estrogen via aromatase. Given the expression of aromatase in human osteoblasts [62] and the common finding of osteopenia and osteoporosis in men with aromatase deficiency [44, 45], it is likely that aromatase has a significant contribution to bone structure in men. There is however limited data on the selective effects of aromatase on bone structure regardless of gender, with the majority of data coming from the overexpression of aromatase in mouse animal models. The overexpression of human aromatase in mouse osteoblasts led to animals with increased overall trabecular BMD, increased cortical BMD, increased cortical thickness, and a reduction in the number of osteoclasts [63]. A different study looked at the effect of global overexpression of human aromatase in mice and found that these animals had increased trabecular BMD, yet cortical bone effects were not analyzed in this study [64]. Confounding these results, however, was the simultaneous overall increase in testosterone and estrogen levels in these mice, making it hard to differentiate between the local effect of aromatase in the bone rather than the global effect of increased sex steroids. Overall, the specific role of aromatase on bone metabolism is an area that needs further study.

Taken altogether, testosterone likely has both direct effects on the bone via signaling through the androgen receptor in osteoblasts and osteocytes as well as indirect effects on the bone via aromatase activity in osteoblasts. Androgen receptor signaling in osteoblasts contributes to trabecular bone formation whereas androgens prevent osteocyte-mediated, age-related trabecular bone loss. Indirectly testosterone affects the bone through local conversion to estrogen by aromatase. This leads to both increased cortical and trabecular bone. This is not completely surprising as aromatase activity should increase local estrogen levels, and male estrogen receptor knockout mice have similarly decreased trabecular and cortical bone development and maintenance [65, 66].

## 4. The Effect of Male Hypogonadism on Bone Quality and Fracture Risk

### 4.1. What Is the Correlation between BMD and Fracture Rate to Circulating Levels of Testosterone, SHBG, and Estrogen?

The precise role of testosterone in the maintenance of bone health and conversely the contribution that low testosterone has to the development of male osteoporosis are still not entirely clear. A number of observational trials have been done to investigate potential risk factors for osteoporosis in men. One of the largest is the *osteoporotic fractures in men study* (MrOS), which followed thousands of men over the age of 65 in Sweden, the United States, and Hong Kong for an average of 4.5 years (Table 2). The initial results of the Sweden cohort of MrOS found that free testosterone levels were positively correlated with BMD in the hip, femur, and arm but not the lumbar spine. Lower levels of free testosterone were also correlated with increased fracture risk [67]. Estrogen levels were positively correlated with BMD in all locations including the lumbar spine [67]. However, further data analysis using multivariate analysis in the same cohort later found that only bioavailable estrogen (BioE2) and SHBG but not testosterone were independently associated with fracture risk [68]. The Hong Kong cohort of the MrOS trial found that only BioE2 was significantly associated with BMD, whereas the levels of SHBG and free testosterone had a nonsignificant correlation with BMD [49]. In terms of fracture risk, neither testosterone nor estrogen levels were predictive of risk, though patients with both low testosterone and low estrogen were at the greatest risk of fracture overall suggesting a combined role of both hormones [49].

The MrOS study also had a North American study group looking at American men over the age of 65, but its results differed slightly from the Hong Kong and Swedish cohorts. Estrogen levels, but not testosterone, exhibited a significant correlation with BMD with the lowest BioE2 levels exhibiting the highest loss of BMD over the study period. SHBG was also negatively associated with BMD and perhaps reflects the free testosterone availability, as SHBG is the major serum-binding partner for testosterone. Finally, men with a combination of low free testosterone and low BioE2 and also men with high SHBG had the highest overall risk of fracture [50]. The overall results of the MrOS suggested that BioE2 is definitively associated with both BMD and fracture risk in elderly men. SHBG is also associated with fracture risk and very likely associated with BMD, while free testosterone is not clearly associated with BMD but may have some role in fracture risk in elderly men. It is important to note that the study population in MrOS, regardless of the country of original, reflects a spectrum of hypogonadal states from normal to below normal. This is likely a true reflection of the events associated with age-related testosterone decline in men and allows for extrapolations for predicted effects on bone quality in elderly men with a range of testosterone levels from normal to true hypogonadism.

A number of studies similar to MrOS have also provided conflicting data on the role of testosterone in male bone health. One study looking at a subgroup from the Framingham cohort looked at the fracture risk in men that were hypogonadal (serum testosterone < 3 ng/mL) as compared to eugonadal men. Men with the highest estradiol levels had the highest BMD, and those with the lowest estradiol levels had the highest risk of hip fracture. There was no difference in BMD or hip fracture risk in hypogonadal men compared to eugonadal men, suggesting no significant role of testosterone in bone health [69]. This role of estrogen and lack of a role of testosterone supports the findings of the MrOS study. Other studies looking for correlations between the development of male osteoporosis and testosterone levels give conflicting results. Australian men in the Dubbo study (>60 years old) were found to have decreased BMD associated with low BioE2 and testosterone levels, but only low testosterone and not estrogen was associated with increased fracture risk, particularly at the hip [70]. Results from the Tromsø study failed to demonstrate any correlation between changes in BMD and estrogen or testosterone levels in men, though high SHBG was significantly associated with lower BMD but was noted to have little role on its overall variance based on predictive models [71]. Finally, the MINOS study once again found that only estrogen, and not lower levels of testosterone, was positively associated with BMD in elderly men. They did note that hypogonadal men had increased rates of falls and markers of bone resorption [72].

### 4.1.1. Summary of Conclusions regarding Testosterone Levels and Bone Quality in Elderly Men

Overall, the findings from these studies suggest that low BioE2 and high SHBG, possibly correlating to a low bioavailable testosterone, both contribute to low BMD in men. In terms of fracture risk, low BioE2, low free testosterone, and high SHBG may all be associated with increased fracture risk. The more prominent role for testosterone in actual fracture risk compared to BMD may be related to testosterone's independent role in muscle strength and physical performance in men as increased muscle weakness would predispose to a high rate of falls potentially leading to fracture [73]. Indeed, a number of smaller studies have found that elderly men with osteoporotic fractures had statistically significant lower levels of testosterone as compared to age-matched controls [74–79] while other studies did not find any association between testosterone levels and fracture risk [80–82]. The risk of falls in elderly men is also associated with lower testosterone levels [83]. The largely variable data on the role of testosterone in BMD and fracture risk may be related to the fact that the studies look at total body testosterone levels which may not necessarily reflect regional testosterone levels within the bone and localized testosterone metabolism. One study using a subgroup within the MrOS trial demonstrated that androgen metabolites but not total testosterone correlated positively with BMD, suggesting that increased local androgen metabolism enhances BMD [84]. With the overall conflicting data on whether testosterone is associated with increased fracture risk in elderly men, it may be useful to look at data from men with prostate cancer treated with androgen deprivation therapy. One study with over 50,000 participants found that androgen deprivation therapy either through gonadotropin-releasing hormone agonist or orchiectomy increased the relative risk of fracture by 1.54 and 1.45, respectively, and when metastatic disease was excluded, the relative risk of fracture in men on a gonadotropin-releasing hormone agonist was still RR 1.37 [82]. A number of other studies have found similar results in men undergoing androgen deprivation therapy for prostate cancer [85–87]. Overall, with the data from observational studies suggesting a role of testosterone in osteoporotic fractures in men combined with proven increased fracture risk in men undergoing androgen deprivation, it seems likely that low testosterone is indeed associated with fracture risk in older men.

### 4.2. Bone Mineral Density, Bone Turnover, and Response to Testosterone Therapy in Young Hypogonadal Men

While these previous studies enrolled large numbers of elderly men, they tend to not look specifically at hypogonadism, but rather overall testosterone levels and how it relates to BMD and fracture risk. For example, in the MrOS study, only men in the lowest testosterone quartile examined would possibly have testosterone levels low enough to meet a clinical diagnosis of hypogonadism. Additionally, the few subjects that would meet the criteria for hypogonadism likely had late onset hypogonadism related to age-related testosterone decline, which is a distinct entity from primary or secondary hypogonadism in younger males [88–90]. Therefore, while useful in suggesting a role of testosterone in BMD and fracture risk, these large observational trials do not directly address the possible role of hypogonadism itself in BMD and fracture risk. There are a number of smaller studies that have compared BMD of hypogonadal men to age-matched controls. These studies demonstrate decreased BMD in hypogonadal males of all ages, particularly in the lumbar spine [91–100].

## 5. Comparison of Bone Quality between Hypergonadotropic and Hypogonadotropic Hypogonadism in Men

### 5.1. Does Bone Density Differ in Primary versus Central (Pituitary and Hypothalamic) Hypogonadal States?

Interestingly, there is some evidence that men with secondary or central hypogonadism (hypogonadotropic hypogonadism) in fact have lower baseline BMD compared to men with primary hypogonadism (hypergonadotropic hypogonadism). Primary hypogonadism is associated with marked elevations of FSH and LH, whereas pituitary or hypothalamic dysfunction results in inappropriately low levels of these two hormones [101–104]. This difference in BMD is somewhat counterintuitive as many people with primary hypogonadism such as Klinefelter's syndrome would be hypogonadal even during pubertal bone development which can cause an overall decrease in the development of peak bone mass [105], and pituitary dysfunction commonly presents after puberty is completed. Assuming there is no bias within these studies for the frequency of prepubertal hypogonadism, the reason for men with secondary hypogonadism to have lower BMD compared to those with primary hypogonadism is not clear. One could speculate that LH or FSH may have protective effects on bone which would be lost in secondary hypogonadism, yet studies have found that FSH stimulates osteoclast activity [106], and in both women and men, higher FSH levels are correlated with decreased BMD [107–110]. Similarly, higher levels of LH seem to be negatively correlated with BMD in women [111] and men [110]. Therefore, a lack of FSH or LH in secondary hypogonadism does not explain a more significant loss of BMD compared to men with primary hypogonadism.

Primary hypogonadism arising from testicular failure also differs from secondary hypogonadism in that additional testes-derived hormones may be specifically lacking, as compared to hypogonadism arising from pituitary/hypothalamic dysfunction. It may be that there are specific factors produced by the gonads that inhibit bone formation, which would then be absent in primary hypogonadism, explaining why men with secondary hypogonadism seem to have more significant declines in BMD as compared to men with primary hypogonadism. However, known gonad-specific hormones, such as inhibin A and insulin-like factor 3, are actually known to positively contribute to BMD, making this explanation unlikely [51, 112]. Further research should be done to confirm if men with secondary hypogonadism do indeed have worse BMD compared to those with primary hypogonadism and why this may be the case.

### 5.2. Are There Clinical Differences between Fracture Risk, Fracture Site, and Degree of Bone Loss between Primary and Central Hypogonadism?

The differences in BMD between primary hypogonadism and secondary hypogonadism suggest that the type of hypogonadism may affect the overall risk of osteoporosis and fragility fracture. Currently, there is a limited amount of data on the effects of different types of hypogonadism on BMD and fracture risk. There are several unique patient populations in both primary hypogonadism (Klinefelter's syndrome, testicular failure from surgery, or chemotherapy) and secondary hypogonadism (opiate abuse, prolactinomas) for which there is data on BMD and fracture risk.

Individuals with primary hypogonadism due to Klinefelter syndrome consistently present with lower BMD compared to that of age-matched controls [113–119], though two smaller studies did not demonstrate any difference in BMD [98, 120]. The decreases in BMD were most typically seen in the lumbar spine and femoral neck. The effect of orchiectomy is less well studied, but patients receiving orchiectomy have higher rates of osteoporosis and osteopenia compared to population controls, and low testosterone levels in these patients were significantly associated with lower BMD [121, 122].

In men with central hypogonadism from pituitary prolactinomas (and the resulting repression of FSH and LH), BMD is frequently found to be reduced to either the osteopenic or osteoporotic range (55% overall) [123–125], with an associated increase in vertebral fracture risk [126]. Interestingly, it was the lack of estrogen, but not testosterone, that was associated with decreased BMD in men with prolactinomas [123, 124]. Patients with idiopathic hypogonadotropic hypogonadism have been found to have universally lower BMD compared to age-matched controls, though no data for possible increased fracture risk was found [94, 95, 98, 104, 116, 127, 128]. Chronic opiate use can also lead to central hypogonadism, and patients on long-term opiates have higher rates of osteopenia and osteoporosis, decreased BMD, and increased fracture risk [129–131]. However, there are multiple potential contributors to decreased BMD and increased fractures in this population, though lower BMD was found to be associated with lower testosterone levels in one study [131]. Other causes of hypogonadism and their potential effects on BMD and fracture risk have not yet been studied. Overall, there is limited data on how specific types of male hypogonadism contribute to BMD and fracture risk, though there is a significant trend for men with hypogonadism to have decreased BMD.

## 6. Treatment Options and Responses in Male Hypogonadal Osteoporosis

### 6.1. What Is the Benefit of Testosterone Replacement on BMD and Fracture Risk in Men with Osteoporosis and Hypogonadism?

Treatment for symptomatic hypogonadal males to improve their symptoms and enhance BMD has been recommended in North America by the Endocrine Society and appears in their 2010 clinical guidelines [132]. Additionally, the 2012 Endocrine Society Osteoporosis in Men guideline also recommends the use of testosterone therapy in men with symptomatic low testosterone who are at high risk of fracture [133] though this should be done in combination with a medication with a proven antifracture effect such as a bisphosphonate. It has been well known that testosterone treatment in hypogonadal males has beneficial effects on BMD, but unfortunately to date, no studies have adequately assessed its clinical impact on fracture risk [132, 133]. Despite the known ability of testosterone to improve BMD in hypogonadal males, testosterone treatment is not recommended to enhance BMD unless they truly have symptomatic hypogonadism. The reasoning at this time is that the potential risks of testosterone therapy (including acne, erythrocytosis, prostate abnormalities, formulation-specific adverse effects, and potential negative sleep apnea and cardiovascular effects) outweigh the benefit to BMD enhancement [132].

There have been a number of small, randomized controlled studies evaluating the effect in men of testosterone treatment on the bone, regardless of underlying testosterone levels, which generally demonstrated improvements to BMD. Hoppéa et al. [134] reviewed 14 of these randomized controlled trials (RCT) of testosterone treatment in males, all of which looked at BMD at the lumbar spine and femoral head, without fracture risk outcomes. Regarding the lumbar spine BMD, only 5 of the 14 showed significant increases, with the remaining showing nonsignificant (6 of 14) or no (3 of 14) differences after therapy. Considering the femoral head, measureable gains were detected in 9 of 14, yet most were nonsignificant differences. These results must be interpreted with caution due to the clear limitations of these studies, including small study sizes (*n* = 30–223), variable follow-up (6–36 months), and great heterogeneity in study population characteristics including such key considerations as age, baseline androgen levels, and dosing/choice of testosterone delivery. Despite these limitations and variations in outcome results, the authors concluded that there was sufficient evidence to suggest, at the least, a benefit in lumbar spine BMD with testosterone therapy. The Endocrine Society came to the same conclusion and incorporated this into their 2010 guideline publication regarding testosterone treatment in hypogonadal males [132].

Since this 2013 review by Hoppéa et al., there have been additional studies in specific male populations that have provided further evidence for the potential benefit of testosterone treatment on lumbar spine and femoral BMD increases. Permpongkosol et al. [135] published a 2016 observational study of testosterone treatment in 120 late-onset hypogonadal males with a mean age of 65.6 years, representing the decline in gonadal testosterone in older men. These participants were treated with intramuscular testosterone injections for 5 to 8 years, the longest study to date. Significant increases in BMD at both the lumbar and femoral neck sites were detected. Interestingly, there was a differential rate of BMD response to therapy between the hip and the spine: significant BMD increases at the femoral neck were not detected until after 48 months of treatment, as opposed to 24 months for significant changes at the lumbar spine. This difference between changes in the femoral neck and spine may be explained by the different contribution of cortical and trabecular bone in these two regions. Vertebrae are comprised of approximately 75% trabecular bone, whereas the femoral head is only 50% trabecular bone [136]. This relatively greater effect of testosterone on trabecular bone structures such as the spine would also be keeping with the greater effect of testosterone on trabecular bone in mouse models. In terms of testosterone side effects, PSA, prostate volume, and hematocrit were found to significantly increase with treatment.

Wang et al. [137] published a two-year open label RCT of 186 hypogonadal males aged >60 with osteoporosis at baseline. The three study arms included oral low dose testosterone treatment, oral standard dose testosterone treatment, and placebo. BMD was assessed intermittently and significant increases in the BMD at the lumbar spine and femoral neck became apparent after 6 months and 12 months in standard dose and low dose testosterone treatment, respectively. These gains remained significant throughout the remainder of the 24-month study. Prior to 18 months, the standard dose treatment gains were significantly greater than the lower dose, but after 18 months of therapy, there was no significant difference between the two groups. Prostate size and PSA levels were monitored but showed no significant differences between study groups.

Bouloux et al. [138] published a one-year double-blinded RCT in 2013 involving 322 hypogonadal males aged ≥50. The four study arms included testosterone therapy with oral low dose, intermediate dose, and high dose testosterone treatment versus placebo. Significant BMD increases were detected the earliest (12 months) and over dispersed body sites (lumbar spine, total hip, trochanter, and intertrochanteric) only for the highest testosterone doses. In contrast, at the 12-month point, the intermediate dose group demonstrated significant BMD increases only for trochanter and intertrochanteric sites. Adverse effects were not analyzed.

A final study by Rodriguez-Tolra et al. [139] is a two-year prospective treatment study involving testosterone therapy in 50 hypogonadal males aged ≥50. Patients were treated with topical testosterone gel for the first 12 months, followed by testosterone intramuscular injection for the remaining 12 months. It is not rationalized why a 12-month period of topical treatment followed by a 12-month period of intramuscular treatment was used. Significant improvements were found in lumbar spine BMD at both 12 and 24 months, total hip and trochanter at 24 months only, and no significant changes for the femoral neck. Of note, hematocrit and PSA levels increased significantly from baseline over the 24 months of treatment.

Although these more recent studies provide further evidence for the anabolic effects of testosterone replacement on lumbar BMD, and also demonstrate improvements of BMD at the femoral site, one great limitation is the lack of clinical determination of fracture rate. Also, the variable rates for adverse effects of testosterone leave the issue of patient risk benefit still up for debate. While testosterone replacement is known to potentially cause erythrocytosis as well as growth of metastatic or subclinical prostate cancer [132], the risk of testosterone therapy in men with heart failure, obstructive sleep apnea, or lower urinary tract symptoms has recently been called into question [140]. Furthermore, controversy remains over the potential risk of testosterone therapy on cardiovascular mortality, though a clear reproducible cardiovascular risk has yet to be demonstrated [140]. There remains the need for larger, longer, multicenter RCTs that evaluate both the change in BMD and fracture incidence for testosterone treatment in hypogonadal males. In addition, subgroup analysis is needed to characterize the relationship of benefit with severity of testosterone deficiency, degree of baseline BMD loss, and the dose of testosterone replacement.

### 6.2. What Is the Role for Bisphosphonate Treatment in Male Hypogonadal-Related Osteoporosis?

The indications for bisphosphonate treatment in males are the same as for females. The 2012 male Osteoporosis Guidelines from the Endocrinology Society has recommended bisphosphonate, or the alternative Denosumab, and also Teriparatide in select populations, to improve BMD and fracture risk for all men aged 50 and older with a spine or hip fragility fracture, osteoporosis, or osteopenia with a high calculated fracture risk [133]. Regardless of whether males are hypogonadal or not, if they fulfill any of these above indications, treatment is recommended.

The benefit for bisphosphonate therapy in the hypogonadal male population is largely extrapolated from these benefits observed in the general male population. There have been a number of studies looking at bisphosphonate treatment for those individuals undergoing androgen deprivation therapy for prostate cancer, which show significant BMD protection during treatment [141–144]. However, it would be difficult to justify extrapolating this data to hypogonadal males with established BMD deficits. A single small (*n* = 22, age 29–69) RCT considered the effect of bisphosphonate treatment in a purely hypogonadal population of men, with proven osteoporosis or osteopenia and long-standing hypogonadism on testosterone replacement over three years [145]. Their treatment arms consisted of Alendronate versus placebo for one year followed by Alendronate in both for the next two years. Within 1 year, significant improvements in BMD occurred at both the lumbar and femoral neck sites. Following the transition to equal Alendronate treatment, lumbar BMD appeared to plateau with no significant improvement in BMD while the femoral neck BMD continued to improve. It is difficult to draw conclusions from a small single center study like this, but as described in the sections to follow, some of the male osteoporosis RCTs did subgroup analysis of low testosterone groups and found equal benefit [146, 147]. Additional larger RCTs in hypogonadal populations are needed to not only confirm the benefits of bisphosphonate treatment but to help quantify its impact and how it compares to male osteoporosis in general.

Gradually over time, RCTs have emerged in the general male population and demonstrate clear benefits of bisphosphonate therapy on BMD and fracture risk [148, 149]. There have been a number of male bisphosphonate RCTs since the beginning of 2000 that have demonstrated a unanimous improvement in BMD as well as a number of them showing a benefit in fracture risk reduction [146, 147, 150–153]. Regardless of the bisphosphonate used, or 1 versus 2 years of follow-up, all trials found significant improvement in lumbar and femoral BMD measurements over the 1-2 years of study. Fractures were also reduced with bisphosphonate therapy, with significant reductions in radiographic vertebral fractures [147, 150, 151], and significant reduction in both vertebral and nonvertebral radiographic fractures [153].

Considering male hypogonadism and bisphosphonate responses by the bone, few studies performed testosterone level subgroup analysis. Orwoll et al. [147] found no difference in lumbar BMD improvements between normal and low testosterone subgroups but did not comment on femoral BMD or fracture incidence. Boonen et al. [152] found a greater degree of BMD improvement in males with lower baseline testosterone.

### 6.3. What Evidence Supports Testosterone and Bisphosphonate Combination Treatment for Male Osteoporosis?

The Endocrinology Society, in their 2012 guidelines, recommends combination therapy with bisphosphonate, or equivalent options, and testosterone replacement only in symptomatic hypogonadal males with indications for pharmacological treatment [133]. There have been no specific RCTs published looking at dual therapy in this population; rather the recommendation stems from the known benefit of both studied in isolation. The impact of the two head-to-head and in combination is not known. Thus, symptomatic hypogonadal males with spine or hip fragility fracture, osteoporosis, or osteopenia with a high calculated fracture risk have indication for testosterone treatment by virtue of their hypogonadal symptoms and have indication for bisphosphonates by virtue of their BMD and fracture risk.

### 6.4. What Is the Evidence for Denosumab or Teriparatide Treatment in Male Osteoporosis?

There have been no RCTs of Denosumab or Teriparatide therapy involving hypogonadal males with osteoporosis or osteopenia. There have been some studies showing the benefit of Denosumab and Teriparatide on BMD in the male population in general [154–158]. Teriparatide has also been observed to show significant reduction in vertebral fractures in males [159]. Denosumab has only shown a significant reduction in vertebral fractures when used in males receiving androgen deprivation therapy [160]. As a result of these findings, the Endocrinology Society has supported the use of Denosumab in the context of androgen deprivation therapy, as an alternative to bisphosphonate therapy in males with indications for treatment [133]. The efficacy of Denosumab and Teriparatide in the hypogonadal male population is yet to be studied.

## 7. Conclusions

Testosterone has a clear direct effect on bone health. Testosterone signaling stimulates osteoblasts to form trabecular bone and helps osteocytes prevent trabecular bone loss. This leads to the decreased BMD and increased fracture risk seen in men with both primary and secondary hypogonadism. Testosterone also has indirect effects on bone through its aromatization to estrogen via aromatase. The role of testosterone in the bone health of elderly men is less clear but likely reduces fracture risk and may potentially contribute to BMD and bone health via aromatization, as estrogen is clearly associated with both fracture risk and BMD in elderly men. Bisphosphonates should be first-line therapy in the treatment of male hypogonadism-related osteoporosis, with the consideration for the addition of testosterone replacement therapy. Other pharmacological therapies specifically for male osteoporosis secondary to hypogonadism have yet to be studied.

## Figures and Tables

**Table 1:** All cause prevalence of osteoporosis by age in men and women (from Kanis [2]).

Age	50–60	60–70	70–80	80–90	>90
Male	0.6%	1.7%	4.3%	10.4%	22.6%
Female	3.4%	8.5%	19.2%	37.3%	61.3%

**Table 2:** Comparison of BMD and fracture risk associations of sex steroids and SHBG from large studies.

		Fracture risk									BMD								
		Testosterone			Estrogen			SHBG			Testosterone			Estrogen			SHBG		
Study	Characteristics	H	V	O	H	V	O	H	V	O	H	V	O	H	V	O	H	V	O
CHAMP	Observational study 958 Australian men over 70 5-year follow-up	N	–	N	N	–	N	Y	–	Y	Y	–	–	Y	–	–	Y	–	–

Dubbo	Observational study 609 Australian men over 60 Average follow-up of 5.8 years	Y	N	N	N	N	N	–	–	–	–	N^∗^	N^∗^	–	Y^∗^	Y^∗^	–	Y^∗^	Y^∗^

LASA	Observational study 623 Dutch men over 55 Follow-up 3–6 years	–	–	N	–	–	Y	–	–	–	Y	–	–	Y	–	–	–	–	–

Tromsø	Observational study 3564 Norwegian men and women aged 25 to 84 (avg age 60) Average follow-up of 6.5 years	–	–	–	–	–	–	–	–	–	–	–	Y^∗^	–	–	Y^∗^	–	–	Y

Framingham	Prospective cohort study 405 men aged 68 to 96 Hypogonadal men (testosterone < 3 ng/mL) versus eugonadal men	–	N	N	–	Y	Y	–	–	–	–	–	–	–	–	–	–	–	–

mROS Sweden	Observational study 3014 Swedish men aged 69–80 Average follow-up of 3.32 years	N	N	N	Y	Y	Y	Y	N	Y	–	–	–	–	–	–	–	–	–

mROS US	Observational study 1234 men over 65 Average follow-up of 4.6 years	–	–	–	–	–	–	–	–	–	N	–	–	Y	–	–	Y	–	–

mROS US	Observational study 1436 American men over 65 Average follow-up of 4.7 years	N	–	N	N	–	Y	Y	–	Y	–	–	–	–	–	–	–	–	–

mROS Hong Kong	Observational study 1498 men over 65 from Hong Kong Average follow-up of 4 years	N	–	N	N	–	Y	N	–	N	N	–	N	Y	–	Y	N	–	N

Rotterdam	Prospective case-control study 179 men over 55 from Rotterdam Average follow-up of 6.5 years	–	–	–	–	N	–	–	N	–	–	–	–	–	Y^∗^	–	–	N^∗^	–

EPIC-Oxford	Prospective cohort study 155 men from Oxford avg age 51 6-year follow-up period	–	–	N	–	–	Y	–	–	N	–	–	–	–	–	–	–	–	–

Y denotes statistically significant association; N denotes no association; – indicates not studied. H = hip, V = vertebral, and O = other sites not including hip or vertebral. ∗ represents analysis was only univariate and not multivariate.

## Additional Points

### Key Points

(1) Female reference populations for bone mineral densities remain an acceptable standard for males aged ≥50, yet there is an increasing worldwide use of male reference populations. (2) The prevalence of male osteoporosis and hypogonadal-related osteoporosis increases with age yet remains lower than females of similar ages. (3) Male hypogonadism results in declines of bone density and is correlated with increased fracture risk, yet estrogen plays the largest role in bone loss and fracture risk in older men. (4) Hypogonadotropic hypogonadism appears to correlate with lower bone quality than hypergonadotropic hypogonadism for unclear reasons. (5) Testosterone replacement therapy can effectively reverse hypogonadism-induced bone loss in men, yet bisphosphonates are considered first-line treatment for established osteoporosis in asymptomatic males. (6) Testosterone signaling through the androgen receptor promotes trabecular bone formation in osteoblasts and inhibits trabecular bone loss through osteocytes but has a largely unknown effect on osteoclasts.
